# Anticipated facilitators and barriers for long-acting injectable antiretrovirals as HIV treatment and prevention in Vietnam: a qualitative study among healthcare workers

**DOI:** 10.1186/s12879-024-10352-w

**Published:** 2024-12-25

**Authors:** My T. Dang, Yen N. Le, Sarah Naz-McLean, Nhung T. T. Vo, Phuong T. Do, Linh T. T. Doan, Nhan T. Do, Mai T. Nguyen, An H. Phan, Eric J. Dziuban, Ramona Bhatia, Lisa Cosimi, Huong T. T. Phan, Todd M. Pollack

**Affiliations:** 1 Department of Medicine, Beth Israel Deaconess Medical Center, Boston, USA; 2 The Partnership for Health Advancement in Vietnam, Hanoi, Vietnam; 3 Division of Global HIV and TB, Center for Global Health, U.S. Centers for Disease Control and Prevention, Hanoi, Vietnam; 4 Division of Infectious Diseases, Brigham and Women’s Hospital, Boston, USA; 5 Vietnam Administration of HIV/AIDS Control, Ministry of Health, Hanoi, Vietnam; 6 Division of Global HIV and TB, Center for Global Health, U.S. Centers for Disease Control and Prevention, Atlanta, USA; 7 The Partnership for Health Advancement in Vietnam (HAIVN), Beth Israel Deaconess Medical Center (BIDMC), 6th Floor, 27 Hang Bai Street, Hanoi, Vietnam

**Keywords:** Healthcare worker, HIV, Implementation, Long-acting injectable antiretrovirals, Prevention, Treatment, Vietnam

## Abstract

**Background:**

Long-acting injectable antiretrovirals (LAI-ARVs) for HIV prevention and treatment have been demonstrated in clinical trials to be non-inferior to daily oral medications, providing an additional option to help users overcome the challenges of daily adherence. Approval and implementation of these regimens in low- and middle-income settings have been limited.

**Method:**

This study describes the anticipated barriers and facilitators to implementing LAI-ARVs in Vietnam to inform future roll-out. From July to August 2022, we conducted 27 in-depth interviews with healthcare workers and public health stakeholders involved in HIV programs at national, provincial, and clinic levels across four provinces in Vietnam. The interviews followed a semi-structured questionnaire and were audio recorded. Data were analyzed using a rapid thematic analysis approach to identify facilitators and barriers to the adoption of LAI-ARVs.

**Results:**

In total, 27 participants from 4 provinces were interviewed including 14 (52%) men and 13 (48%) women. Participants median age was 48 years and they had 11.5 years of experience with HIV services and programs. Perceived user-level facilitators included the greater convenience of injectables in comparison to oral regimens, while barriers included the increased frequency of visits, fear of pain and side effects, and cost. Clinic-level facilitators included existing technical capacity to administer injections and physical storage availability in district health centers, while barriers included lack of space and equipment for administering injections for HIV-related services, concerns about cold chain maintenance for LAI-ART, and workload for healthcare workers. Health system-level facilitators included existing mechanisms for medication distribution, while barriers included regulatory approval processes and concerns about supply chain continuity.

**Conclusion:**

Overall, participants were optimistic about the potential impact of LAI-ARVs but highlighted important considerations at multiple levels needed to ensure successful implementation in Vietnam.

**Clinical trial number:**

Not applicable.

**Supplementary Information:**

The online version contains supplementary material available at 10.1186/s12879-024-10352-w.

## Background

Antiretroviral (ARV) drugs are highly effective for both the treatment and prevention of human immunodeficiency virus (HIV). The World Health Organization (WHO) recommends a dolutegravir-containing daily oral fixed-dose antiretroviral therapy (ART) regimen for people living with HIV (PLHIV) due to high rates of viral suppression, excellent tolerability and safety, and high barrier to resistance [1–3]. However, to achieve the desired efficacy, these oral ART regimens require consistent and lifelong adherence to the recommended dosing schedule, which may be challenging for some individuals due to pill fatigue, side effects, stigma, or other circumstances [4, 5]. ARVs are also highly effective for persons without HIV in preventing HIV transmission. Oral pre-exposure prophylaxis (PrEP) containing tenofovir disoproxil fumarate is 99% effective at preventing sexual transmission of HIV when taken as directed [6]. Oral PrEP regimens also have challenges related to acceptability and adherence due to risk perception, side effects, stigma, and variability of individual routines [7].

Long-acting injectable antiretrovirals (LAI-ARVs) provide an additional option that may help users overcome adherence challenges to daily oral medications. For HIV treatment, a two-drug intramuscular regimen of cabotegravir (CAB) and rilpivirine (RPV) has been demonstrated in clinical trials to be non-inferior to daily oral ART for maintenance of viral suppression [8–11]. For people without HIV, long-acting injectable PrEP (LAI-PrEP) consisting of cabotegravir delivered by intramuscular injection has been demonstrated to be superior to oral PrEP for the prevention of sexually acquired HIV among men who have sex with men (MSM), transgender women (TGW), and cisgender women [12, 13].

Following successful clinical trials, in 2020, Health Canada became the first regulatory agency worldwide to approve Cabenuva (cabotegravir/rilpivirine) as the first LAI-ART regimen for the treatment of HIV-1, and in 2021, the United States Food and Drug Administration (US FDA) approved Apretude (Cabotegravir Long-Acting) as the first LAI-PrEP regimen for HIV prevention. Both injectable regimens have since been approved by the US FDA, the European Medicines Agency, and the Australian Pharmaceutical Benefits Scheme. Despite these milestones, approval and implementation of these medications have predominantly been limited to high-income settings, while approval in low- and middle-income country (LMIC) settings has been slower. Generic formulations of these medications, while in early planning stages for LAI-PrEP, are not currently available.

In July 2022, the WHO issued guidelines recommending Cabotegravir Long-Acting (CAB-LA) as an additional prevention choice for people at substantial risk of HIV infection. In November 2022, Zimbabwe became the first African country to approve CAB-LA, which has since been approved in several other LMIC settings. In 2024, Zambia became one of the first LMICs to offer CAB-LA outside of study settings [14]. However, implementation of both LAI-PrEP and LAI-ART in LMICs has predominantly been limited to clinical trials or demonstration projects [15]. Prior research has indicated that barriers to the programmatic roll-out of LAI-ARVs in LMICs may include a lack of feasibility studies, cost-effectiveness data, funding, and political will [16].

Vietnam is a middle-income country in Asia with an estimated 250,000 PLHIV. As of 2023, while the HIV prevalence in the general population was 0.3%, the burden of HIV infection was concentrated among key populations, with the highest prevalence reported among MSM (12.5%), people who inject drugs (9.1%), and sex workers (2.5%) [17]. Vietnam has made significant progress towards achieving the UNAIDS 95-95-95 goals: In 2023, 88% of PLHIV knew their status, 80% of PLHIV who knew their HIV status were on ART, and 98.3% of PLHIV on treatment had a suppressed viral load. The health system for delivering ART in Vietnam has been well developed, with 534 clinics providing ART across the country’s 63 provinces and 93% of all PLHIV who are on treatment receiving their ARVs under Social Health Insurance (SHI) coverage. In addition, there were 219 public and private health facilities providing free PrEP services to 67,183 users in 29 provinces by 2023 [18].

Despite Vietnam’s established HIV prevention and care system and the achievement of national ART and PrEP targets in 2023 [18], further efforts are needed to optimize engagement and retention in prevention and care services in order to reach “the last mile” and realize the goal of ending AIDS by 2030 [19, 20]. While ART adherence rates among PLHIV in Vietnam are generally high, studies have shown that certain populations, such as people who use drugs, face challenges with ongoing ART adherence and retention in care [21, 22]. Additionally, daily oral PrEP persistence in Vietnam has been reported at 43% in one study and 33% in another study at 12 months [20, 23]. The availability of LAI-ARV formulations has the potential to benefit users by providing them with an additional choice to overcome the challenges of daily oral regimens [15]. LAI-ARVs may also help close program gaps by reaching populations that are difficult to engage through traditional oral regimens. Given the limited data on LAI-ARVs implementation in LMICs in general, in the Asian region, and in Vietnam in particular, we conducted a study to better understand the barriers and facilitators to the introduction of LAI-ARVs in Vietnam. Study findings will be used as a foundation to advocate for and inform the preparation for LAI-ARVs roll-out in the country.

## Methods

### Study design

The data presented in this manuscript were collected as part of a larger mixed-methods study evaluating the feasibility and acceptability of novel long-acting antiretrovirals for HIV treatment and prevention in Vietnam. The overall study consisted of one-time structured interviews with people living with and at risk of HIV and in-depth interviews with healthcare workers and public health officials involved in HIV program implementation. In this paper, we present the results of the healthcare worker component of the study, conducted in July and August 2022 in four provinces of Vietnam (Hanoi, Hai Phong, Ho Chi Minh City, and Binh Duong) with a focus on LAI-ARVs. These provinces were chosen based on their high HIV prevalence and receipt of President’s Emergency Plan for AIDS Relief (PEPFAR)/ U.S. Centers for Disease Control and Prevention (U.S. CDC) program support, as well as because they included a range of geographical and economic conditions. The study was implemented by the Vietnam Administration of HIV/AIDS Control (VAAC), in cooperation with Beth Israel Deaconess Medical Center (BIDMC) and the U.S. CDC.

### Study participants, sampling and recruitment

The participants recruited for the in-depth interviews were key stakeholders involved in HIV program implementation at the national, provincial, and clinic levels. For all participants, inclusion criteria were being 18 years of age or older and able to provide informed consent. Clinic-level participants were healthcare workers eligible to prescribe ART and PrEP with at least 6 months of experience directly delivering HIV prevention and care services to patients. Provincial-level participants were public health managers with at least 6 months of experience in designing and implementing HIV prevention and treatment programs at the provincial level. National-level participants were public health officials working at the Ministry of Health (MOH) in departments related to HIV policy, regulation, and programming, such as VAAC, Department of Health Insurance, Drug Administration of Vietnam, and Vietnam Administration of Medical Services.

VAAC and BIDMC generated a list of potential participants including the medical doctors working at public ART/PrEP sites in the target provinces, the public health officials responsible for the HIV program at the provincial CDCs and officials from relevant MOH departments as identified by VAAC. This list was then purposely sampled based on professional role, institutional placement (e.g., public vs. private clinic, provincial Center for Disease Control), and gender to include diverse representation. Those interested in participating were scheduled for face-to-face interviews. Recruitment was continued until thematic saturation was reached.

### Interview guide and procedures

Participants were invited to participate in a one-time study visit that lasted approximately one hour and was conducted at a place of their choice. Participants received 200,000 VND (approximately 8.7 USD) for their participation in the study. Following informed consent, the study procedures included: (1) a brief demographic questionnaire, (2) a short introduction to LAI-ARVs, read aloud by study staff, and (3) an in-depth qualitative interview.

The interviews followed a semi-structured guide with open-ended questions. The guide was adapted from a questionnaire used in a previous study [24]. It was designed to gain participants’ perspectives on the potential facilitators and barriers to implementing LAI-ARVs across three domains: (1) technical or clinical, (2) logistical, and (3) regulatory or legal, and included questions on clinical characteristics affecting users’ acceptability, licensing and importation of new agents, cost and health insurance coverage, storage, administration, and other logistical considerations. The questionnaire was initially developed in English, translated into Vietnamese, and then back-translated into English to ensure content consistency.

### Data collection and data analysis

The data collected were analyzed using a rapid thematic analysis approach [25, 26]. Prior to the interviews, a coding matrix was developed in Microsoft Excel^®^ to capture barriers and facilitators across the three domains. The codebook was developed based on known barriers and facilitators identified through existing literature and was adapted as needed throughout the interview process to capture emerging themes. The interviews were audio recorded. At least two members of the study team joined each interview; while an interviewer asked questions, an analyst observed and captured notes. Immediately following the interview, the analyst and interviewer discussed key themes and illustrative quotes, coded the notes and quotes into the coding matrix, and listened to the audio recording to ensure accuracy.

The qualitative data presented in this manuscript were organized according to user, clinic and health system level. For each level, we present participant perspectives collected and consolidated from the key domains on anticipated facilitators and barriers to implementing LAI-ARVs in Vietnam.

## Results

### Participant characteristics

In total, 27 participants were interviewed, including clinic-level healthcare providers (*n* = 15), provincial-level health managers (*n* = 6), and national-level public health officials (*n* = 6). Participant characteristics are shown in Table 1.

**Table 1:** Characteristics of 27 participants from 4 provinces in Vietnam (2022), by health system level

	Clinic level (*n* = 15)	Provincial level (*n* = 6)	National level (*n* = 6)	Total (*N* = 27)
Participants by provinces				
*Hanoi*	3	-	6	**9**
*Hai Phong*	4	2	-	**6**
*Binh Duong*	4	2	-	**6**
*Ho Chi Minh city*	4	2	-	**6**
Age in years, median (IQR)	39.5 (18)*	50 (5)	46 (11)	**48 (14)**
Years of experience in HIV program, median (IQR)	6 (8)*	18 (3)	14.5 (9)	**11.5 (12)**
Sex				
*Male*,* n (%)*	7 (47%)	5 (83%)	2 (33%)	**14 (52%)**
*Female*,* n (%)*	8 (53%)	1 (17%)	4 (67%)	**13 (48%)**
Self-reported awareness of LAI-ARVs, n (%)**	12 (80%)	6 (100%)	3 (50%)	**21 (78%)**

IQR: Interquartile Range; LAI-ARVs: Long-acting injectable antiretrovirals

* 1 missing data

** % who reported being “somewhat aware” or “aware” of LAI-ARVs for treatment and prevention

Approximately equal numbers of men and women participated in the interviews (52% men, 48% women). The median age was 48 years (Interquartile Range - IQR: 14). Participants had a wide range of experience with delivering HIV services and programs, with a median of 11.5 years of experience (IQR: 12). Participants who were HIV program managers at the provincial and national levels had been participating in HIV programs for a longer period of time, ranging from 14.5 to 18 years, compared to doctors working at the clinic level with a median of 6 years of experience. When asked about awareness of LAI-ARVs prior to their participation in the study, 80% of clinic-level participants and 100% of provincial-level participants reported being aware of LAI-ARVs, compared to only half of the national-level participants.

### Main findings

#### Anticipated facilitators and barriers at the user level

Participants discussed their perceptions of how LAI-ARVs might impact their patients’ care and whether the new injectable regimens would be well-received by patients. They highlighted several key facilitators and, overall, believed that LAI-ARVs would be highly acceptable to users as they may offer greater convenience in comparison to oral daily regimens. First, they perceived that LAI-ARVs would reduce the psychological burden of taking daily oral pills, which require regular and lifelong adherence (for ART). The majority of participants thought that users who struggle with daily adherence would potentially benefit from LAI-ARVs. Second, they agreed that LAI-ARVs provided additional options for users who have difficulties tolerating oral regimens (e.g., patients who struggle to swallow pills) or whose job or lifestyle make it challenging to take medications daily. Third, participants perceived that LAI-ARVs may offer increased confidentiality and decreased stigma, as users would not have to store medication bottles and set specific times to take pills, reducing the chances of their ARV use being noticed by others.

*“The injectable drug will help a lot because clients don’t have to remind themselves to take pills over and over. Moreover*,* it will make life easier for clients. Taking medicine along to work is less convenient*,* compared to taking an injection every one to two months.”*

– *Health Manager, Binh Duong*.

*“Injectable medications are advantageous for patients whose job requires frequent travel*,* or who have irregular working hours*,* like working in shifts*,* because sometimes they are too busy to take oral drugs or shift work is not fixed. Therefore*,* it is appropriate for them to use injectable drugs.”*

– *Clinic Doctor, Hai Phong*.

Nevertheless, participants also highlighted perceived barriers to acceptability and uptake of LAI-ARVs at the user level. The most salient concern was the increased frequency of medical appointments, from once every 90 days (the current standard of care in Vietnam, given that ART and PrEP pills can be dispensed as 90-day refills) to once every 30–60 days for LAI-ART and once every 60 days for LAI-PrEP. Participants noted that while injectable regimens may ease the burden related to daily pill-taking, they could potentially increase the burden to users if more frequent medical appointments are required. This may affect their retention, medication refills, and adherence. Participants also anticipated that fear of pain and side effects may also be additional barriers to user acceptability. Although the out-of-pocket cost of LAI-ARVs in Vietnam is still unknown, participants highlighted that cost may be another important barrier. For LAI-ART, participants noted that oral ART regimens are currently covered by social health insurance and speculated that users may not be willing or able to pay for LAI-ART if it is more expensive. For LAI-PrEP, participants speculated that PrEP clients may be more willing to pay out-of-pocket for the added convenience compared to ART clients, as they perceived that their PrEP clients, on average, were of higher socioeconomic status.

“*If the price is reasonable, more people can afford it. If it is too expensive, few clients still can make it given our patients have different financial conditions…About 30%, meaning one-third of PrEP clients, can pay for the medications; this rate in ART patients is lower, less than one-third of the total current patients can do so.”*

– *Health Manager, Ho Chi Minh city*.

Overall, participants felt that communication between healthcare staff and users would be one of the most important factors influencing user acceptance of the new long-acting regimens. Healthcare workers suggested that information about LAI-ARVs, such as efficacy, side effects, and benefits, should be widely disseminated to doctors so they are equipped to provide this information to their patients. They believed that if users could weigh the potential benefits of LAI-ARVs, they would be highly accepting of the new injectable long-acting regimens.

#### Anticipated facilitators and barriers at the clinic level

At the clinic level, participants discussed how factors such as human resources and storage facilities could support the delivery of LAI-ARVs. For example, participants highlighted that nurses already have the technical capacity to administer injections as this is part of all nurses’ standard training. Further, some participants felt that on-site physical storage and refrigerators would be accessible to store injectable drugs because ART/PrEP clinics are integrated into district health centers that provide vaccination and other injection services.

“*Nurses are familiar with injection practice because they are not only [HIV] out-patient clinic staff but also nurse residents [of the district health center].“*

– *Clinic Doctor, Hai Phong*.

Despite the availability of these resources, other participants acknowledged that the lack of medical equipment to store cold chain medications could be a potential barrier due to the temperature requirements for LAI-ART containing rilpivirine. They also felt that further investment in clinics would be needed to administer injections, as clinics that offer ART and PrEP services are currently equipped to administer oral medications only. Participants anticipated that clinical encounters to administer injections would be more complex than the care that is currently provided during normal refill visits for oral medication. They noted that implementing LAI-ARVs at clinics would necessitate additional staff, equipment, and infrastructure, such as beds, rooms, and anaphylaxis response kits, among others. Participants also highlighted that injection visits may be more frequent and longer in duration than oral medication refill visits, all of which could increase the workload for healthcare workers at clinics. Given those barriers, healthcare workers recommended that clear guidelines for the implementation, administration, and management of LAI-ARVs at the clinic level are needed to advocate for infrastructure investments, human resources mobilization, and training to properly administer and manage clients receiving injections at clinics.

*“In fact*,* it does not matter for a clinic to roll out the injections because physicians and nurses are available at the clinics*,* and also a management system as stated in a Circular on adverse event management issued by the Ministry of Health recently. So it’s not a problem for health clinics in general. For HIV outpatient clinics*,* they do not practice injections*,* but only dispense medicines every 3 or 6 months. So*,* if we roll out this*,* we will need to invest more for the [HIV outpatient] clinics. Certainly*,* health staff there will need to be trained about the new regimen*,* so that they can be well-prepared.”*

– *Health Manager, Hai Phong*.

“*There must be a cold storage refrigerator located in the injection room managed by the pharmacy department with specific procedures on management and monitoring. … If the medications need to be kept cold, what temperature is required?… If they need to be kept at 0–4 degrees, it will be costly to purchase a cold storage refrigerator. However, if we roll out the novel medications, it’s compulsory to equip it. Pharmacists need to check the temperature of the refrigerator at least twice a day to ensure the temperature requirement for cold storage.”*

– *Clinic Doctor, Hanoi*.

#### Considerations on LAI-ARVs implementation at the health system level

Participants believed that, given the existing number of ART and PrEP users and the high quantity of medications managed nationally, Vietnam’s health system is well prepared to manage the delivery of LAI-ARVs. During the interview, a health manager at the national level stated that Vietnam has a reliable logistics system for the delivery of ART and PrEP medications, which has been functioning well for many years. This available system can be adapted for managing and implementing LAI-ARVs in the future. In addition, Vietnam’s HIV program previously has experience with rolling out injectable drugs for prevention programs, such as injectable regimens used in the management of substance use disorder; thus, introducing new long-acting injectable medicines is achievable for the health system in Vietnam.

*“[The health system] can manage thousands of pills*,* the injectable drug is not a big deal …This is not the first type of injectable drugs in the HIV/AIDS program. You know*,* there are injectable drugs within the HIV/AIDS prevention area*,* such as an injectable regimen for addiction treatment*,* which is also a long-acting drug*,* either implanting or injectable drug.”*

– *Health Manager, Hanoi*.

Healthcare workers also acknowledged that there are several health system barriers to the implementation of LAI-ARVs in Vietnam. Firstly, they highlighted the lengthy process of obtaining regulatory approval for medications in the country. This could be challenged by the limited information on LAI-ARVs implementation in other countries within the region, which is typically used to support the regulatory approval of new medications. In particular, there is currently a lack of price comparison with neighboring countries in the region, making it challenging for the MOH to approve the price of LAI-ARVs in the Vietnam market. Secondly, most participants at provincial and clinic levels expressed concerns about the continuity of the supply chain for LAI-ARVs because disruptions may affect treatment and prevention outcomes. They described that the bidding mechanism at the national level has, at times, resulted in temporary supply chain interruptions for the current oral regimens due to delays in the central procurement procedure. As a result, participants anticipated that the supply chain for LAI-ARVs may encounter similar challenges as oral drugs. Clinic and provincial level participants expressed concern that disruptions in the LAI-ARVs supply would create difficulties for users as they would need further consultations from doctors to switch to alternative oral regimens. They highlighted that changes to regimen, schedule, or dosage may cause confusion for clients, thereby affecting their treatment outcomes.

*“Supply chain is always a critical issue. Like the current oral drug*,* which SHI has covered since 2019*,* there have always been disruptions in the supply chain in the first few months of the year. For example*,* the current distributors of the current oral drugs can’t distribute medicines as planned to all clinics. Therefore*,* maintaining the supply of drugs for months for our clients*,* who are using the service*,* is also a challenge.”*

– *Health Manager, Binh Duong*.

*“I’m only concerned about the interruption of the drug supply. For instance*,* I’m not sure if it’s possible and easy to switch from injectable to oral medications if there is a drug supply shortage when patients receive injections. I need to know about it to advise them accordingly. Even now*,* many types of oral ARV drugs are not available. We must switch between different types to make them suitable for patients. Those familiar with injections will ask for injections; they won’t want to switch back and forth between oral and injectable medications.”*

– *Clinic Doctor, Ho Chi Minh city*.

*“In order to circulate the drugs in Vietnam*,* the first step is to register for circulation through the Drug Administration of Vietnam*,* Ministry of Health. This is the most difficult process. Firstly*,* to get approval for a new drug*,* it is necessary to have a clinical application of safety and efficacy in the country where the marketing authorization is granted… It takes 2–3 years to evaluate this application… To circulate and then distribute in the Vietnamese market*,* it is necessary to declare the drug price to set the price for the product on the Vietnamese market… The most common problem here is to have prices of neighboring countries to form price comparison*,* which is a foundation for the Ministry of Health of Vietnam to approve fixed prices in Vietnam.”*

– *Health Manager, Hanoi*.

Most participants recommended that a pilot of LAI-ARVs was needed to gain practical experience of LAI-ARVs implementation. Results from the pilot would provide real-world evidence on the acceptability of LAI-ARVs, medication effectiveness, and safety among Vietnamese users, which could be used as inputs to a clinical evaluation dossier for registration approval.

## Discussion

**Fig. 1:**
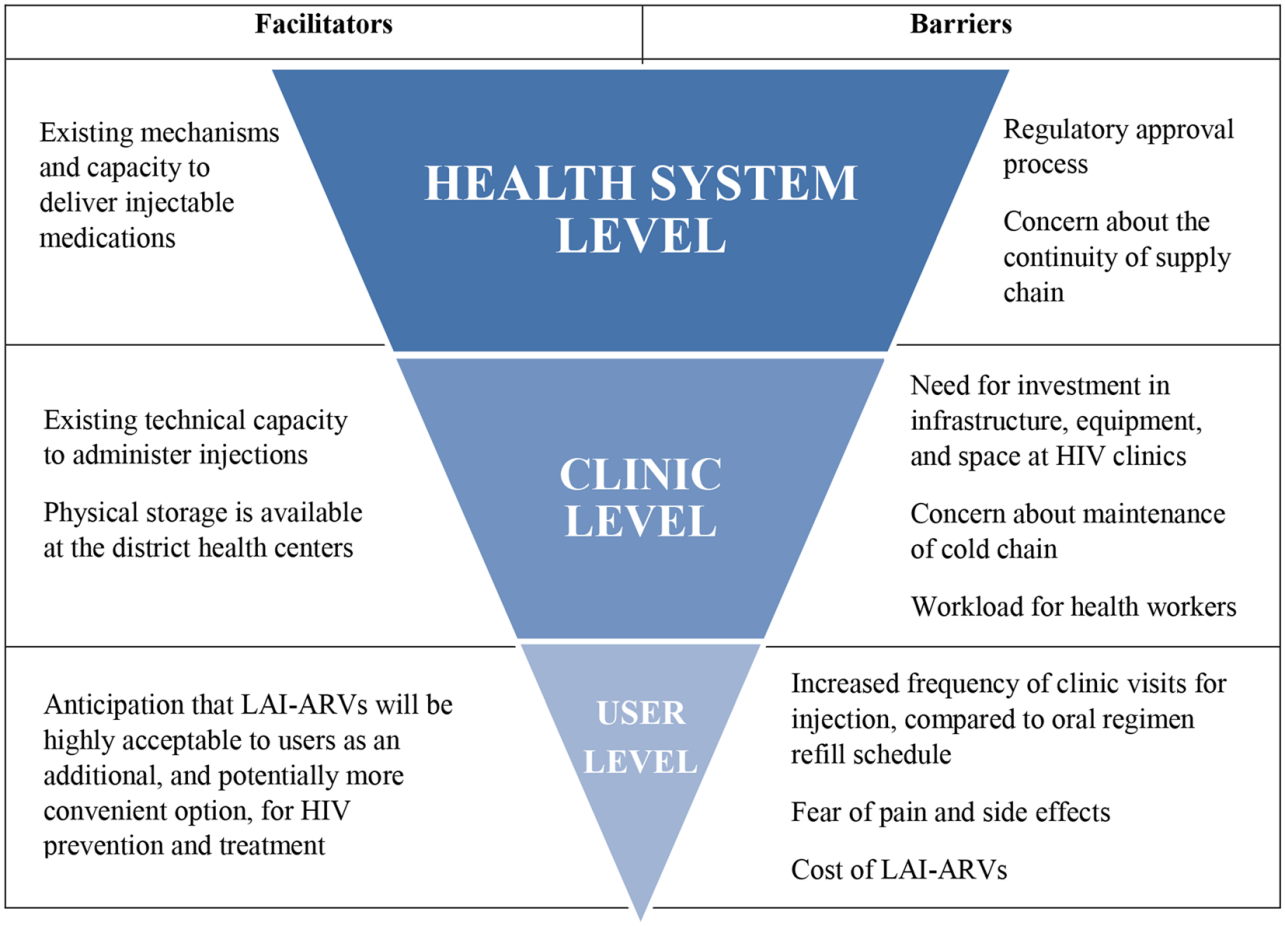
Anticipated facilitators and barriers to the implementation of long-acting antiretrovirals in Vietnam. *Legend*: Fig. 1 summarizes the anticipated facilitators and barriers to the implementation of long-acting antiretrovirals from the perspective of healthcare workers organized according to the user, clinic, and health system level. Participating healthcare workers were recruited from four provinces in Vietnam and included medical doctors working at public HIV clinics, public health officials responsible for the HIV program at the provincial level, and officials from within the Ministry of Health

Our study identified anticipated facilitators and barriers to implementing LAI-ARVs in Vietnam at the user, clinic, and health system levels from the perspectives of experienced and diverse health providers and managers (Fig. 1). The results showed that healthcare workers in Vietnam are optimistic that LAI-ARVs could be a valuable option to help their clients overcome the current challenges with oral ARV regimens. This finding is not only reflected in other studies on healthcare provider perceptions of long-acting regimens [27], but is also consistent with published data on acceptability from potential ART and PrEP users [28, 29]. Future work with potential LAI-ARV users in Vietnam and other regional LMIC settings may be considered to better understand patient acceptability and to inform demand creation approaches prior to the introduction of these new regimens in Vietnam. Most of our study participants acknowledged that the primary benefit of the LAI regimens would be to ease their clients’ burden of daily adherence. They felt that LAI regimens would, therefore, be particularly useful for their clients who are struggling to take their daily oral medications. Currently, LAI-ART regimens (i.e., CAB/RPV) have been approved for use in those who have already attained viral suppression, but emerging evidence from case series shows promising results in offering LAI-ART to those who struggle with adherence and have a high HIV viral load [30–32].

In general, our findings on anticipated barriers to implementing LAI-ARVs in Vietnam are similar to those identified in other studies from LMICs [24, 33–35]. Such potential barriers include uncertainty about patients’ ability to adhere to injection visits, the lack of experience in implementing LAI-ARVs in LMICs, unknown or high cost, complicated regulatory approval processes, concerns about supply chain interruptions, the need for appropriately trained personnel, and readiness at the clinic level in terms of staffing, equipment, and infrastructure. Nevertheless, findings from our study and similar studies in LMIC settings [36] show that healthcare workers believe these barriers can be overcome and that implementing LAI-ARVs in their setting is feasible and beneficial.

Our findings highlight several important resource considerations at the clinic level. First, participants anticipated that patient encounters would likely be longer and more frequent for injectable regimens – every 30–60 days, compared to the 90-day refill visits required for oral regimens. They also noted the added complexity of injection appointments, such as requiring additional time for monitoring patients and being prepared with anaphylaxis response kits. Jointly, these factors may have important impacts on staff workload, underscoring the importance of planning human resource needs accordingly. Second, there was no clear consensus among participants on the current readiness of clinics to provide cold storage that would meet rilpivirine requirements for storing LAI-ART (2°to 8 °C or 35.6° to 46.4 °F) [37]. Some participants were confident in the existing capacity of clinics, noting that clinics integrated within district health centers would have access to appropriate equipment. However, others expressed concerns that clinics lacked the necessary infrastructure to refrigerate medications on-site as would be required for CAB/RPV LAI-ART. Prior research and programmatic efforts in Vietnam have demonstrated that the health system has both the regulatory and cold chain infrastructure (2–8 °C) in place to store and administer injectable medications at the central, provincial, and district levels [38, 39]. However, experience from the roll-out of COVID-19 vaccines did find challenges related to a lack of sufficient cold chain infrastructure at the lower levels of the health system [40]. This, along with the lack of concordance between stakeholders in our study, highlights the potential benefits of demonstration or pilot projects to better inform the rollout of these regimens in Vietnam. Further, healthcare providers and managers noted that clear guidelines on the administration and implementation of LAI-ARVs from the central level would play a key role in ensuring smooth implementation at the clinic level. These official guidelines from the central government would allow provincial departments of health, health facility leaders, and district health centers to coordinate and mobilize resources needed, including staff and infrastructure investment, to administer the injectable regimens at clinics.

Cost was highlighted as potentially being a major barrier to the implementation and uptake of LAI-ARVs in LMICs [33]. First, in relation to the regulatory approval process, there is currently a lack of price comparison from nearby countries, which was highlighted by national-level stakeholders as a crucial factor needed to determine the price of LAI-ARVs within Vietnam. Second, once approved for use in the country, study participants anticipated that the cost of LAI-ARVs would be a significant barrier to access for those who may benefit the most from the new regimens because the current oral medications are offered free through programs or SHI coverage and it was unclear whether LAI-ARVs would be covered by SHI. Government subsidies or co-payment assistance may be needed [24] to improve access. Participants of our study strongly recommended that LAI-ARVs be covered by the country’s SHI program in order to make LAI-ARVs more accessible and affordable in Vietnam. Currently, most PLHIV receive oral ART through SHI with a co-payment of less than two USD per month. However, participants felt that getting LAI-ARVs onto the list of drugs covered by SHI could be challenging and would take time. For example, very few participants thought LAI-PrEP could be covered by SHI because even oral PrEP is not covered and is currently offered free by the government only with support from donor programs. Most also believed that PLHIV would not be willing or able to pay out-of-pocket for LAI-ART, because currently 93% of PLHIV on treatment depend on SHI for oral ART [18]. Participants thought it would be more likely that PrEP users could afford to pay for LAI-PrEP because they typically are better educated, have better financial situations, and may only use PrEP during periods of HIV risk.

Overall, most study participants believed that making LAI-ARVs available to users as an additional choice for HIV treatment and prevention would improve the HIV program in Vietnam. Most agreed that clear implementation guidance and pilot preparation, including obtaining a supply of the new medications for a small-scale pilot, would be critical next steps. The Vietnam National HIV Guidelines, issued under Decision 5968/QD-BYT in December 2021 [41], are currently being revised. It is anticipated that LAI-ART (CAB/RPV) and LAI-PrEP (CAB-LA) will be included in the revised guideline. This will offer a legal framework for the implementation of LAI-ARVs in Vietnam. Following the inclusion of the new regimens in the national guidelines, a pilot program can be organized to collect real-world data on the use of LAI-ARVs in the Vietnam setting to inform the development of documents needed for regulatory approval in the country. Recently, during the International AIDS Society Conference in July 2023, the Vietnam Ministry of Health committed to be one of the first countries in Asia to start the roll-out of CAB-LA [42, 43].

### Limitations

Our study has three main limitations. First, the clinic and provincial participants were recruited from provinces receiving PEPFAR/U.S. CDC support. These provinces and sites may differ in terms of investment in healthcare worker training or healthcare infrastructure compared to provinces outside of PEPFAR support. Our results, therefore, may not reflect the opinions of all healthcare workers in the country. In addition, we were not able to recruit healthcare managers at the provincial level in Hanoi and so did not have responses from all provincial representatives across the four study provinces. Second, participants in our study only included medical doctors providing HIV prevention or treatment, as well as public health managers and officials. Nurses and community healthcare workers play an important role in providing ART and PrEP services to clients, and future research may include these groups to better understand potential barriers and facilitators at the clinic level. Lastly, it is important to note that while the self-reported awareness of LAI-ARVs was high among participants, conversations with participants about barriers and facilitators were largely hypothetical in nature as LAI-ARVs are not yet approved or available in Vietnam. Future work may focus on reevaluating the barriers and facilitators that may emerge and be identified through real-world implementation of these medications in Vietnam.

## Conclusion

This study has provided valuable insights into the anticipated facilitators and barriers to implementing LAI-ARVs for HIV treatment and prevention in Vietnam, as perceived by healthcare workers at various levels of the health system. The findings underscore a general optimism among healthcare providers regarding the potential benefits of LAI-ARVs in improving adherence and offering greater convenience for users, particularly those who struggle with daily oral regimens. However, HIV programs may consider addressing several critical barriers identified through this study to ensure successful implementation. These include concerns about the increased frequency of clinic visits, cost, and the need for adequate infrastructure and trained personnel to manage injections and cold storage requirements. Additionally, regulatory hurdles and potential supply chain disruptions pose significant challenges.

Healthcare workers highlighted the necessity for clear guidelines and pilot programs to gather real-world data and inform the rollout of LAI-ARVs. Given the existing strengths in Vietnam’s HIV program, such as the established logistics system for ART and PrEP and experience with other injectable regimens, there is a solid foundation upon which to build the introduction of LAI-ARVs. However, attention to resource allocation, staff training, and ensuring an uninterrupted supply chain will be crucial.

To move forward, these identified barriers may be addressed through targeted interventions, including policy advocacy for regulatory approvals, securing funding for infrastructure and cold chain requirements, and ensuring that LAI-ARVs are affordable and accessible, possibly through integration into the SHI system. By addressing these challenges, Vietnam can enhance its HIV prevention and treatment programs, ultimately contributing to the global goal of ending AIDS by 2030. Future research may consider assessing patient acceptability and the practical challenges encountered during the implementation of LAI-ARVs to further refine and optimize their rollout.

## Electronic Supplementary Material

Below is the link to the electronic supplementary material.

Supplementary Material 1

## Data Availability

The datasets used and/or analyzed during the current study are available from the corresponding author upon reasonable request.

## Abbreviations

ART: Antiretroviral therapy
ARV: Antiretroviral
BIDMC: Beth Israel Deaconess Medical Center
CAB: Cabotegravir
CAB-LA: Cabotegravir Long-Acting
HIV: Human immunodeficiency virus
LAI-ARVs: Long-acting injectable antiretrovirals
LAI-PrEP: Long-acting injectable PrEP
LMIC: Low- and middle-income country
MOH: Ministry of Health
MSM: Men who have sex with men
PEPFAR: The U.S. President’s Emergency Plan for AIDS Relief
PLHIV: People living with HIV
PrEP: Pre-exposure prophylaxis
RPV: Rilpivirine
SHI: Social health insurance
TGW: Transgender women
U.S. CDC: United States Centers for Disease Control and Prevention
US FDA: United States Food and Drug Administration
VAAC: Vietnam Administration of HIV/AIDS Control
WHO: World Health Organization

## References

[1] World Health Organization. Updated recommendations on first-line and second-line antiretroviral regimens and post-exposure prophylaxis and recommendations on early infant diagnosis of HIV: interim guidelines. Supplement to the 2016 consolidated guidelines on the use of antiretroviral drugs for treating and preventing HIV infection. Geneva, WHO. 2018. https://iris.who.int/bitstream/handle/10665/277395/WHO-CDS-HIV-18.51-eng.pdf?sequence=1&isAllowed=y. Accessed 1 October 2023.

[2] World Health Organization. Update of recommendations on first- and second-line antiretroviral regimens. Geneva, WHO., 2019. https://iris.who.int/bitstream/handle/10665/325892/WHO-CDS-HIV-19.15-eng.pdf. Accessed 1 October 2023.

[3] Kandel CE, Walmsley SL. Dolutegravir - a review of the pharmacology, efficacy, and safety in the treatment of HIV. Drug Des Devel Ther. 2015;9:3547–55. 10.2147/DDDT.S84850. Published 2015 Jul 7. 26185421 10.2147/DDDT.S84850 PMC4500604

[4] Margaret A, Chesney J. Factors affecting adherence to antiretroviral therapy. Clinical Infectious Diseases, 2000;30(2):S171–76. 10.1086/313849. 10.1086/313849 10860902

[5] Katz IT, Ryu AE, Onuegbu AG, Psaros C, Weiser SD, Bangsberg DR, et al. Impact of HIV-related stigma on treatment adherence: systematic review and meta-synthesis. J Int AIDS Soc. 2013;16(3). 10.7448/IAS.16.3.18640 PMC3833107 24242258

[6] Division of HIV Prevention, National Center for HIV, Viral Hepatitis, STD, and TB Prevention. Centers for Disease Control and Prevention, Effectiveness of Prevention Strategies to Reduce the Risk of Acquiring or Transmitting HIV. 2022. https://www.cdc.gov/hiv/risk/estimates/preventionstrategies.html. Accessed 12 April 2024.

[7] Sidebottom D, Ekström AM, Strömdahl S. A systematic review of adherence to oral pre-exposure prophylaxis for HIV - how can we improve uptake and adherence? BMC Infect Dis. 2018;18(1):581. 10.1186/s12879-018-3463-4. 30445925 10.1186/s12879-018-3463-4 PMC6240194

[8] Orkin C, Arasteh K, Górgolas Hernández-Mora M, et al. Long-Acting Cabotegravir and Rilpivirine after Oral Induction for HIV-1 Infection. N Engl J Med. 2020;382(12):1124–35. 10.1056/nejmoa1909512. 32130806 10.1056/NEJMoa1909512

[9] Swindells S, Andrade-Villanueva JF, Richmond GJ, et al. Long-Acting Cabotegravir and Rilpivirine for Maintenance of HIV-1 Suppression. N Engl J Med. 2020;382(12):1112–23. 10.1056/nejmoa1904398. 32130809 10.1056/NEJMoa1904398

[10] Orkin C, Bernal Morell E, Tan DHS, et al. Initiation of long-acting cabotegravir plus rilpivirine as direct-to-injection or with an oral lead-in in adults with HIV-1 infection: week 124 results of the open-label phase 3 FLAIR study. Lancet HIV. 2021;8(11):e668–78. 10.1016/s2352-3018(21)00184-3. 34656207 10.1016/S2352-3018(21)00184-3

[11] Durham SH, Chahine EB. Cabotegravir-Rilpivirine: The First Complete Long-Acting Injectable Regimen for the Treatment of HIV-1 Infection. Ann Pharmacother. 2021;55(11):1397–409. 10.1177/1060028021995586. 33593093 10.1177/1060028021995586

[12] Landovitz RJ, Donnell D, Clement ME, et al. Cabotegravir for HIV Prevention in Cisgender Men and Transgender Women. N Engl J Med. 2021;385(7):595–608. 10.1056/nejmoa2101016. 34379922 10.1056/NEJMoa2101016 PMC8448593

[13] Delany-Moretlwe S, Hughes JP, Bock P et al. Cabotegravir for the prevention of HIV-1 in women: results from HPTN 084, a phase 3, randomised clinical trial [published correction appears in Lancet. 2022;399(10337):1778]. Lancet. 2022;399(10337):1779–1789. 10.1016/s0140-6736(22)00538-4 10.1016/S0140-6736(22)00538-4 PMC9077443 35378077

[14] U.S. Embassy in Zambia, U.S. Government delivers first shipment of Injectable PrEP to Zambia. 2024. https://zm.usembassy.gov/first-shipment-of-injectable-prep-to-zambia/. Accessed 24 April 2024.

[15] Kityo CM, (presenter Paton N). Randomized trial of cabotegravir and rilpivirine long-acting in Africa (CARES): week 48 results. 31st Conference on Retroviruses and Opportunistic Infections, Denver, abstract 122, 2024.

[16] Philbin MM, Perez-Brumer A. Promise, perils and cautious optimism: the next frontier in long-acting modalities for the treatment and prevention of HIV. Curr Opin HIV AIDS. 2022;17(2):72–88. 10.1097/coh.0000000000000723. 35225248 10.1097/COH.0000000000000723 PMC8915989

[17] Joint United Nations Programme on HIV/AIDS (UNAIDS). Country Factsheet: Vietnam. UNAIDS. 2023. https://www.unaids.org/en/regionscountries/countries/vietnam. Accessed 20 November 2023.

[18] Ministry of Health, Issued Decision No 612/QD-BYT on National Plan for HIV/AIDS Control and Prevention in 2024. 2024. https://vaac.gov.vn/upload/tai-lieu/qd-612-ban-hanh-kh-phong-chong-hiv-2024-1.pdf?v=1.0.0. Accessed 8 April 2024.

[19] Hoang NT, Foo TJ, Tran BX, Do NT, Vu GT, Nguyen CT, Pham HQ, Latkin CA, Ho CSH, Ho RCM. Structural barriers for retention of HIV/AIDS patients after initiating antiretroviral therapy in outpatient clinics of Vietnam. AIDS Care. 2022;34(8):992–9. 10.1080/09540121.2021.1929816. 34018428 10.1080/09540121.2021.1929816

[20] Nguyen VTT, Dat VQ, Truc HM, et al. Preference and retention of daily and event-driven pre-exposure prophylaxis for HIV prevention: a prospective cohort in Can Tho city, Viet Nam. BMJ Open. 2024;14:e075976. 10.1136/bmjopen-2023-075976. 38423779 10.1136/bmjopen-2023-075976 PMC10910397

[21] Nguyen PM, Thach AN, Pham XD, Lam AN, Nguyen TNP, Duong CX, Nguyen LV, Nguyen TH, Pham ST, Taxis K, Nguyen T. Prevalence and Determinants of Medication Adherence among Patients with HIV/AIDS in Southern Vietnam. Infect disease Rep. 2021;13(1):126–35. 10.3390/idr13010014. 33562451 10.3390/idr13010014 PMC7931092

[22] Ha TV, Hoffman IF, Miller WC, Mollan KR, Lancaster KE, Richardson P, Zeziulin O, Djoerban Z, Sripaipan T, Chu VA, Guo X, Hanscom B, Go VF. Association between drug use and ART use among people living with HIV who inject drugs in Vietnam, Ukraine and Indonesia: results from HPTN 074. J Subst use. 2022;27(6):648–57. 10.1080/14659891.2021.1989509. 36742268 10.1080/14659891.2021.1989509 PMC9897261

[23] Bui HTM, Giang LM, Chen JS, et al. A Brief Alcohol Intervention (BAI) to reduce alcohol use and improve PrEP outcomes among men who have sex with men in Vietnam: study protocol for a randomized controlled trial. Trials. 2024;25:552. 10.1186/s13063-024-08382-5. 39164770 10.1186/s13063-024-08382-5 PMC11337901

[24] Mantsios A, Murray M, Karver TS, et al. Multi-level considerations for optimal implementation of long-acting injectable antiretroviral therapy to treat people living with HIV: perspectives of health care providers participating in phase 3 trials. BMC Health Serv Res. 2021;21:255. 10.1186/s12913-021-06214-9. 33743684 10.1186/s12913-021-06214-9 PMC7980753

[25] Nevedal AL, Reardon CM, Opra Widerquist MA, et al. Rapid versus traditional qualitative analysis using the Consolidated Framework for Implementation Research (CFIR). Implement Sci. 2021;16:67. 10.1186/s13012-021-01111-5. 34215286 10.1186/s13012-021-01111-5 PMC8252308

[26] Lewinski AA, Crowley MJ, Miller C, et al. Applied Rapid Qualitative Analysis to Develop a Contextually Appropriate Intervention and Increase the Likelihood of Uptake. Med Care. 2021;59(Suppl 3):S242–51. 10.1097/mlr.0000000000001553. 33976073 10.1097/MLR.0000000000001553 PMC8132894

[27] Xavier Hall CD, Smith JC, Driggers RA, et al. PrEParing for long-acting injectable PrEP in the South: perspectives from healthcare providers in Georgia. AIDS Care. 2021;33(6):706–11. https://doi.org/10.1080%2F09540121.2020.1810616. 32838546 10.1080/09540121.2020.1810616 PMC8152107

[28] Slama L, Porcher R, Linard F, et al. Injectable long acting antiretroviral for HIV treatment and prevention: perspectives of potential users. BMC Infect Dis. 2023;23(1):98. 10.1186/s12879-023-08071-9. Published 2023 Feb 17. 36803606 10.1186/s12879-023-08071-9 PMC9936705

[29] Kennedy CE, Zhao T, Vo AV, et al. High acceptability and perceived feasibility of long-acting injectable antiretroviral treatment among people living with HIV who are viremic and health workers in Uganda. AIDS Patient Care and STDs. 2023;316–22. 10.1089/apc.2023.0017. 10.1089/apc.2023.0017 PMC10280193 37294280

[30] Kilcrease C, Yusuf H, Park J, et al. Realizing the promise of long-acting antiretroviral treatment strategies for individuals with HIV and adherence challenges: an illustrative case series. AIDS Res Ther. 2022;19:56. 10.1186/s12981-022-00477-w. 36435793 10.1186/s12981-022-00477-w PMC9701425

[31] Scarsi KK, Swindells S. The Promise of Improved Adherence With Long-Acting Antiretroviral Therapy: What Are the Data? J Int Assoc Provid AIDS Care. 2021;20:23259582211009011. https://doi.org/10.1177%2F23259582211009011. 33902356 10.1177/23259582211009011 PMC8082990

[32] Aadia I. Rana1 et.al, Long-Acting Injectable CAB/RPV Is Superior to Oral ART in PWH With Adherence Challenges. 31st Conference on Retroviruses and Opportunistic Infections, Denver, abstract 212, 2024.

[33] Kityo C, Cortes CP, Phanuphak N, Grinsztejn B, Venter F. Barriers to uptake of long-acting antiretroviral products for treatment and prevention of HIV in Low- and Middle-Income Countries (LMICs). Clinical Infectious Diseases. 2022;75(4):S549–56. 10.1093/cid/ciac752. 10.1093/cid/ciac752 36410377

[34] Mgodi NM, Murewanhema G, Moyo E, Samba C, Musuka G, Dzinamarira T, Brown JM. Advancing the use of Long-Acting Extended Delivery formulations for HIV prevention in sub-Saharan Africa: challenges, opportunities, and recommendations. J Int AIDS Soc. 2023;26:e26115. 10.1002/jia2.26115. 37439069 10.1002/jia2.26115 PMC10338997

[35] Cresswell FV, Lamorde M. Implementation of long-acting antiretroviral therapy in low-income and middle-income countries. Curr Opin HIV AIDS. 2022;17(3):127–34. 10.1097/coh.0000000000000732. 35439787 10.1097/COH.0000000000000732

[36] Carillon S, Laborde-Balen G, Diop M, et al. Implementing long-acting injectable antiretroviral treatments in Senegal: issues, challenges and conditions for introducing them. Qualitative study with healthcare providers and patients. AIDS Care Published online September. 2023;14. 10.1080/09540121.2023.2253506. 10.1080/09540121.2023.2253506 37708454

[37] Vii V, Healthcare. Storage and stability of cabotegravir and rilpivirine suspensions for injection. 2023. https://d201nm4szfwn7c.cloudfront.net/5f95dbd7-245e-4e65-9f36-1a99e28e5bba/3f3384cc-8168-4f0e-a8b7-f523ce3006ec/3f3384cc-8168-4f0e-a8b7-f523ce3006ec_viewable_rendition__v.pdf?medcommid=MED--US-7392#:~:text=The%20recommended%20storage%20range%20for%20rilpivirine%20injection%20is%202%C2%B0,for%20up%20to%206%20hours. Accessed 12 March 2024.

[38] Ministry of Health. Issued Decision No. 1210/QD-BYT on Approving Plan for receipt, storage, distribution and use of Covid-19 vaccines provided by Covax Facility for 2021–2022. 2021. https://thuvienphapluat.vn/van-ban/The-thao-Y-te/Quyet-dinh-1210-QD-BYT-2021-bao-quan-phan-phoi-su-dung-vac-xin-phong-COVID19-2021-2022-465075.aspx. Accessed 13 March 2024.

[39] Ministry of Health. Issued Decision No. 23/2008/QD-BYT on Promulgating the regulation on use of vaccines and medical biologicals in prophylaxis and therapy. 2008. https://thuvienphapluat.vn/van-ban/The-thao-Y-te/Quyet-dinh-23-2008-QD-BYT-Quy-dinh-su-dung-vac-xin-sinh-pham-y-te-trong-du-phong-va-dieu-tri-67983.aspx. Accessed 13 March 2024.

[40] Nguyen VM, Moi F, Boonstoppel L, et al. The cost of delivering COVID-19 vaccines in Vietnam. BMC Health Serv Res. 2024;24:779. 10.1186/s12913-024-11202-w. 38977967 10.1186/s12913-024-11202-w PMC11232236

[41] Ministry of Health. Issued Decision No. 5968/QD-BYT on National Guidance on HIV Care and Treatment. 2021. https://thuvienphapluat.vn/van-ban/The-thao-Y-te/Quyet-dinh-5968-QD-BYT-2021-huong-dan-Dieu-tri-HIV-AIDS-499380.aspx. Accessed 12 December 2023.

[42] Vietnam Administration of HIV/AIDS Control. In: Preparing for CAB-LA introduction in Vietnam. 2023. https://vaac.gov.vn/viet-nam-chia-se-nghien-cuu-thi-diem-thuoc-tiem-prep.html. Accessed 10 December 2023.

[43] Vietnam Administration of HIV/AIDS Control. In: Person-centered PrEP: Framing Vietnam’s approach to integrating PrEP and primary health care. 2023. https://vaac.gov.vn/prep-lay-con-nguoi-lam-trung-tam-dinh-hinh-cach-tiep-can-cua-viet-nam-trong-long-ghep-prep-va-cham-soc-suc-khoe-ban-dau.html. Accessed 10 December 2023.

