# Supplementary material for: Anticipated facilitators and barriers for long-acting injectable antiretrovirals as HIV treatment and prevention in Vietnam: a qualitative study among healthcare workers
Source: BMC Infect Dis. 2024 Dec 25;24:1462. doi: 10.1186/s12879-024-10352-w (PMC11669199; doi:10.1186/s12879-024-10352-w)
Supplement: Supplementary file 1 — Supplementary Material 1 [file 12879_2024_10352_MOESM1_ESM.docx]

## **Appendix 11: Interview Guide for In-Depth Interviews of Health Workers**

**PTID [ ] [ ] [ ] [ ] [ ] [ ]**

**Date:**

**Location:**

**Gender:**

**Age:**

**Job Title:**

**Institution:**

**Years of Experience with HIV:**

*Thank you for taking the time to speak with me today. We appreciate your participation in this project of the* ***Feasibility and acceptability of novel long-acting antiretrovirals for treatment and prevention of HIV in Vietnam****. We are interested in hearing your perspective about the feasibility and potential challenges of rolling-out new ways for HIV clients to take their ART and PrEP, such as by injection. This interview should take no more than one hour. I will be asking you about three main areas of consideration: Clinical/Technical, Logistical, and Regulatory & Payment.*

*Before we begin, I will read you a short description of new long-acting ARVs that have recently been approved and/or recommended for use in other countries.*

***Long-acting injectable PrEP (LA-CAB):*** *A new long-acting injectable PrEP regimen has recently been recommended for use in the United States. The drug regimen contains Cabotegravir (CAB), a novel integrase strand transfer inhibitor (INSTI) and structural analogue of dolutegravir. The regimen is given once every 2 months through intramuscular gluteal injections. The recommended dosing is 3 ml suspension of CAB 600 mg given IM using a 1.5-inch needle. CAB is recommended as an option for all individuals considering PrEP. CAB injections may be especially appropriate for patients with significant renal disease, those who have had difficulty with adherence to oral PrEP and those who prefer injections every 2 months over an oral PrEP dosing schedule. CAB should not be administered to persons with a history of hypersensitivity reaction to cabotegravir. Like oral PrEP, CAB does not protect against other STIs or unintended pregnancies. In large research studies, the new long-acting CAB was more effective than oral daily PrEP in preventing HIV infection. Serious side effects are uncommon. Pain, swelling, and redness at the site of the injection are common after each injection but become less common and severe over time. Before starting on the long-acting injectable version of the medication, individuals may be given the oral daily pill version of the same medication for 4 weeks to make sure there are no allergic reactions or other serious side effects. Due to the long half-life of CAB, individuals who miss doses or discontinue CAB PrEP are at increased risk of acquiring infection with INSTI-resistant HIV. For these reasons, patients discontinuing CAB injections who may be at ongoing risk of sexual and injection HIV exposure should be provided with another effective HIV prevention method during the months following their last injection.*

***Long-acting vaginal ring PrEP (DPV-VR):*** *In January 2021, the WHO recommended the dapivirine vaginal ring (DPV-VR) as an additional prevention choice for women at substantial risk of HIV infection. DPV-VR is a flexible, silicone ring that a woman can insert in the vagina for monthly protection against HIV (show picture). The ring is designed to provide women with a discreet and long-acting option for HIV prevention. It contains the NNRTI (same class as Efavirenz) dapivirine, which is released slowly to reduce the risk of HIV infection locally in the vagina with few effects elsewhere in the body. To properly use the ring, it must be worn inside the vagina for a period of 28 days, after which it should be replaced by a new ring. The ring is made of silicone and is easy to bend and insert. Research studies showed that the monthly vaginal ring reduced women’s risk of HIV infection by around 50 percent with higher levels of protection in women who used the ring most regularly. The ring does not prevent STIs or pregnancy. [SHOW THE IMAGE OF THE VAGINAL RING]*

***Long-acting ART (CAB/RPV):*** *The first long-acting injectable ART was approved in the United States (US) in January 2021. The regimen contains two ARVs - Cabotegravir (CAB) and Rilpivirine (RPV). CAB is a novel integrase strand transfer inhibitor (INSTI) and structural analogue of dolutegravir. RPV is a non-nucleoside reverse transcriptase inhibitor (NNRTI), which was first approved by the US Food and Drug Administration (FDA) in an oral tablet formulation in 2011.* *The regimen is given once a month or once every 2 months through intramuscular gluteal injections. It reduces dosing days per year from 365 with daily oral ART to 6 days with once every 2 month injectable ART. The current formulation requires two 2-ml injections, given via ventrogluteal IM injections with 1½ inch intramuscular needles. CAB and RPV do not have activity against hepatitis B infection and have not been studied in pregnancy. The regimen is recommended to be used as a way to simplify taking ART for people with HIV currently on oral ART with documented viral suppression for at least 3 months****,*** *who have no baseline resistance to either medication, have no prior virologic failures, do not have active hepatitis B infection (unless also receiving an oral hepatitis B active regimen), are not pregnant and are not planning on becoming pregnant, and are not receiving medications with significant drug interactions with CAB or RPV. In large research studies, the new long-acting ART regimen was at least as effective as oral ART in treating HIV. Serious side effects with the injectable regimen are uncommon. Pain, swelling, and redness at the site of the injection are common after each injection but become less common and severe over time. Before starting on long-acting injectable CAB/RPV, individuals would be given the oral daily pill versions for 4-weeks to make sure there are no allergic reactions or other serious side effects. Due to the long half-life of CAB and RPV, individuals who miss doses or discontinue therapy without starting an oral regimen are at increased risk of virologic failure with development of drug resistance. Oral-bridging therapy should therefore be made available for planned missed doses. RPV requires a cold chain for storage and transport.*

*Before we start the interview, do you have any questions about what I just explained?*

Before my introduction, what was your awareness/understanding of the new long-acting injectable ARVs for HIV prevention or treatment (LA-CAB or Long-acting CAB/RPV)?

1. Very aware
2. Aware
3. Somewhat aware
4. Not aware
5. Not sure

Before my introduction, what was your awareness/understanding of the new long-acting vaginal ring for HIV prevention (DPV-VR)?

1. Very aware
2. Aware
3. Somewhat aware
4. Not aware
5. Not sure

**Semi-Structured Interview Questions:**

1. Clinical/Technical issues
   1. What are your initial reactions to the idea of a new HIV regimen for treatment or PrEP that do not have to be taken daily and will be given by injection or vaginal ring?
   2. What role do you think long- acting ARVs may have as part of ART management in Vietnam?
   3. What role do you think long- acting PrEP may have for preventing HIV in Vietnam?
   4. What characteristics of these agents from a clinical standpoint would make them useful for patients in Vietnam?

*🡪 [probe if necessary: dosing schedule, side effects, patient preferences]*

- 1. What characteristics of these agents from clinical standpoint would make you concerned about using them for patients in Vietnam?

*🡪 [probe if necessary: indications / contraindications, dosing, side effects, cost, drug interactions]*

- 1. Do you think your patients/clients in Vietnam would want to take long-acting ARVs when they are available?
     1. Long-acting injectable ART? Why or why not?
     2. Long-acting injectable PrEP? Why or why not?
     3. Long-acting vaginal ring PrEP? Why or why not?

1. Logistics
   1. What other injectable medications or vaccines are being administered at your clinical setting or in Vietnam in general? Are there any challenges/difficulties? How have these been overcome?
   2. What logistical or organizational barriers or challenges do you anticipate when providing injectable ARV agents in the current HIV or PrEP clinics?

*🡪 [probe if necessary: supply chain, storage, staffing]*

- 1. What logistical or organizational barriers or challenges do you anticipate when *providing vaginal ring PrEP in the current HIV or PrEP clinics?*

*🡪 [probe if necessary: supply chain, storage, staffing]*

- 1. What supply chain issues could you anticipate?

🡪 *[probe if necessary: requirement of cold chain for RPV]*

1. Regulatory/Payment
   1. Given what you know about new drug approval in Vietnam, what barriers would you anticipate in getting these new medications approved for use in Vietnam?

- 1. Given what you know, would these new agents be eligible for prescribing under SHI? ART? PrEP? What would be the process to get them covered by SHI? What would be the anticipated barriers?
  2. Do you think patients/client would be willing to pay out of pocket for these new agents for ART? For PrEP? Why or why not?

1. Final questions:
   1. What benefits to public health and the health system could you foresee long-acting ARVs having?
   2. Are you in favor of making long-acting ARVs available for patients in Vietnam, why or why not?
   3. Would you be willing to prescribe/approve long acting ARVs for eligible patients when they are available?
      1. Long-acting injectable ART? Why or why not?
      2. Long-acting injectable PrEP? Why or why not?
      3. Long-acting vaginal ring PrEP? Why or why not?
